# The role of offensive processes and age development for female soccer players’ anticipation

**DOI:** 10.1038/s41598-024-54311-6

**Published:** 2024-03-13

**Authors:** Yizhou Shui, Xiang Che, Yu Zhang, Ning Ma, Jie Li, Xuqun You, Bingjun Wan

**Affiliations:** 1 School of Physical Education, Shaanxi Normal University, Xi’an, 710062 China; 2 Key Laboratory of Behavior and Cognitive Neuroscience of Shaanxi, School of Psychology, Shaanxi Normal University, Xi’an, 710062 China; 3 School of Psychology, Beijing Sport University, Beijing, China; 4 Center for Cognition and Brain Disorders, The Affiliated Hospital, Hangzhou Normal University, Hangzhou, China

**Keywords:** Perceptual-cognitive skills, Pattern anticipation, Offensive process, Female soccer players, Adolescents, Psychology, Human behaviour

## Abstract

Anticipation has been confirmed as a more valid measure for recognizing talented athletes than pattern recall alone. Anticipation of offensive processes in soccer, such as counter attacks and positional attacks, is essential for the sport. Additionally, the anticipation of elements such as the soccer ball, offensive and defensive players may also be affected by varied offensive processes. In this study, we combined anticipation with the pattern recall paradigm to measure the perceptual-cognitive skills of female soccer players across different age groups and offensive processes. Adult (U23) and adolescent (U15) female soccer players were recruited to complete the pattern anticipation task using coach-rated video segments. Our results show that adult female soccer players demonstrated greater accuracy in anticipating locations during positional attacks compared to adolescents, but no significant difference was observed during counter attacks. Furthermore, location anticipation accuracy is higher in all groups towards elements of the soccer ball and offensive players, but not defensive players, during counter attacks compared to positional attacks. These findings suggest that positional attack is the main advantage in perceptual-cognitive skills for adult female soccer players. Additionally, offensive processes and elements should be carefully considered when measuring perceptual-cognitive skills.

## Introduction

Competitive sports are defined by the pursuit of victory through defeating opponents. As such, performance in the sporting arena is strongly correlated with winning or losing, suggesting that sustained success can only be achieved by those with superior abilities. Athletic performance is a strong predictor of outcomes and can significantly impact an athlete’s professional longevity. To enhance their performance, athletes must develop both physical and perceptual-cognitive skills^1^. The skills refer to the ability to identify and acquire environmental information for integration with existing knowledge such that appropriate responses can be selected and executed^2^. Apart from physical fitness, an athlete’s perceptual-cognitive skills within a sport-specific context may also play a crucial role in determining game outcomes^2,3^.

In team sports, perceptual-cognitive skills are increasingly being recognized as crucial indicators of an athlete’s proficiency. These mental skills complement physical abilities, supporting optimal game performance. A review of previous research indicates that expert athletes consistently outperform lesser-skilled and novice athletes on sport-specific cognitive measures^4^. These skills include the ability to recognize and recall complex patterns related to sporting situations^5,6^. Such perceptual-cognitive skills are considered essential for achieving optimal game performance^5^.

The importance of perceptual-cognitive skills in expert soccer performance is well-established. Soccer players must constantly track the movements of teammates and opponents^7^, perceive and identify their rapidly changing positions, make quick and accurate decisions, and execute actions at precise moments^8–10^. These skills have been described as the capacity to detect and process environmental information and integrate it with pre-existing knowledge and motor skills^3,11^.

Among the various methods for measuring perceptual-cognitive skills, the pattern recall task is widely used. This task requires athletes to recall the locations of players presented on a screen and is commonly employed to assess perceptual-cognitive abilities^11^. A review of previous research reveals two main categories of pattern recall tasks: image-based^12,13^ and video-based^14,15^. In pattern recall tasks, after viewing a video segment or image for a specified duration, all presented elements (i.e. players) disappear, and participants are required to recall their locations. The main differences of video-based and image-based pattern recall tasks are whether the game stimuli presented as dynamic video segments or static images. Video-based pattern recall tasks have been shown to be more effective than image-based in distinguishing between experts and non-experts because participants with domain expertise are better able to understand the relationships among the component parts of the pattern^16^. For this reason, video materials were chosen for use in this study.

Pattern recall tasks have been demonstrated to effectively distinguish between team sport players of varying skill levels and developmental stages^11,17^. However, the use of pattern recall tasks has recently come under scrutiny. Van Maarseveen et al.^18^ suggest that pattern recall tasks merely require participants to memorize the present locations of players, making it difficult to couple this information with their future actions. Additionally, pattern recall tasks are suggested to be only a fundamental cognitive process for anticipating future locations of players^19,20^. Furthermore, Kalén et al.^21^ argue that tasks that include anticipation in sport-specific contexts have a better ability to differentiate between higher and lower skilled athletes than tasks without anticipation or not in sport-specific contexts. This approach has been proposed as an alternative method for measuring perceptual-cognitive skills to only pattern recall^20,22^.

The studies mentioned above suggest the relative importance of anticipation in assessing players’ skill levels. Therefore, developing a modified “pattern recall” task that includes anticipation is worth considering to strengthen the identification of talented soccer players. This would involve asking participants to anticipate the future locations of all players based on video segments from soccer games. We refer to this task as pattern anticipation, which differs from only recalling the present location as in pattern recall tasks, but instead requires participants to anticipate players’ future locations. In a similar vein, Farrow et al.^20^ utilized an anticipation task to measure the performance of rugby players at various levels in Line-out situations. Their study provided a preliminary measurement method for anticipating locations in team sports. Building on this work, we developed a task referred to as pattern anticipation in soccer, drawing on the approach used by Farrow et al.^20^.

Pattern anticipation in soccer involves participants anticipating the future locations of elements (ball and all players), after viewing video segments of soccer games. However, some studies have suggested that stimuli background information such as situational probability or contextual information can strongly influence anticipatory behavior. Abernethy et al.^23^ studied the impact of occluded contextual information on experts’ perception in squash. Their findings revealed that experts excel in perceiving both directional and depth information when the display is occluded during the opponent’s hitting action. McRobert et al.^24^, on the other hand, investigated the effect of contextual information in a cricket-batting task. They manipulated high and low context conditions, where participants either viewed their opponent multiple times or responded without prior observation. Using eye tracking data, they found that skilled batters outperformed less skilled ones in anticipation, employed a more effective visual search strategy, and developed more detailed domain-specific representations. In soccer, offensive processes such as counter attacks and positional attacks are one of contextual information can affect team behaviors and the speed at which actions are generated^25^. Therefore, it is important to distinguish between real game offensive processes while examining the pattern anticipation task in soccer.

Varied soccer offensive processes may strongly relate to the team formations of the offensive side. A counter attack occurs when a team launches an offensive immediately after regaining possession of the ball from defending against an opponent’s attack. During this transition, the prior defensive players must shift from defensive to offensive formations, often in an unstructured manner. For offensive players during a counter attack, the risk is relatively low while the chance of scoring is comparatively high^26^ as they face fewer defensive players and have more open space to exploit normally the defensive team in this transition is more disorganized. In contrast, a positional attack is a complex dynamical system in which all players coordinate their spatial positioning^27^. Offensive players during a positional attack face more defensive players in highly structured formations, making it more difficult to anticipate future locations. For example, the positioning of attacking team players may draw an opponent towards the ball, freeing up a teammate elsewhere to receive the next pass. Additionally, for the soccer ball, its motion speed is much higher than that of players whether in a counter attack or positional attack. How the soccer ball moves to exact locations may affect team formation changes for both the offensive and defensive teams^28^. Considering soccer ball perception in varied offensive processes can contribute to enhancing applicability and validity in dynamic game environments.

Therefore, both the elements and offensive processes should be considered when investigating perceptual-cognitive skills. Additionally, incorporating offensive processes into the pattern anticipation task can provide researchers and practitioners with a better understanding of how to use this task to evaluate players’ perceptual-cognitive abilities and make tactical decisions.

Perceptual-cognitive skills are also influenced by the roles of team sports players presented in the stimuli. For instance, experts’ pattern recall abilities vary depending on whether they are recalling offensive or defensive elements. Gorman et al.^29^ found that expert basketball players were more accurate than novices in recalling offensive players but not defensive players. This research underscores the importance of differentiating between elements presented in pattern recall stimuli. Additionally, presented players usually move with team formations, which may also play an essential role. Sherwood et al.^30^ found that expert rugby players performed better than novices on structured stimuli, such as chess openings or attack formations in sports^31^, but not on semi-structured or unstructured stimuli (both rated by coaches). Thus, considering the elements in the pattern anticipation task can provide a novel and well-motivated measure of perceptual-cognitive skills.

Studies have demonstrated that experts possess superior perceptual-cognitive skills compared to novices in areas such as pattern recall^18^. This finding was in line with recognizing the development with the age of players. Waelle et al.^12^ investigated the development of pattern recall abilities in volleyball players aged 6 to 27 years. They found that the perceptual-cognitive skills of youth volleyball players increased throughout adolescence. This result is consistent with findings from Ward and Williams^17^, who observed similar trends among soccer players of varying age groups. For female soccer players, around 15 years old is a key time period to offer their first professional contracts^32^. Therefore, investigating their perceptual-cognitive skills for soccer players of varied age groups is worth taking into consideration. This may provide us additional information for how they develop into elite soccer players.

In addition to investigating the performance of elements and age developement on the pattern anticipation task, it is also important to investigate the role of different offensive processes in this task. Counter attacks and positional attacks are both common offensive processes in soccer. These two processes can result in very different running and passing routes for players during games, which may affect their ability to anticipate future locations^33^. Counter attacks present with fewer players with higher motion speed, in contrast to more players with low motion speed in positional attacks. It is not easy to say which of the offensive processes can be anticipated with high accuracy. Therefore, investigating how these different offensive processes influence performance on the pattern anticipation task is worth considering.

In summary, in the present study, we aim to provide another measurement method of perceptual-cognitive skills in soccer, the pattern anticipation task, which modified previous pattern recall tasks. The applicability of this task in varied offensive processes for female soccer players of varied age groups should be examined. First, we aim to compare the performance of adult and adolescent soccer players on the pattern anticipation task in light of previous findings from pattern recall studies. Gorman et al.^19^ suggested that expert players performed better than novices in recalling offensive players; thus, we hypothesize that adult players anticipate the locations of offensive players better than adolescents but not defensive player anticipating (H1). Additionally, previous studies have not adequately measured the location of the soccer ball, which moves rapidly during passing or shooting and is strongly associated with soccer team behavior and formation^28^. The motion speed of the soccer ball is much higher than that of players and may require high anticipation demand for its movement. To address this issue, we hypothesize that anticipating the location of the soccer ball is more difficult than anticipating the locations of offensive or defensive players (H2).

## Methods

### Participants

According to a power analysis (G*Power; medium effect size, f = 0.25, between-within interaction), a sample of at least 20 participants for each group was needed to achieve at least 0.80 power at α = 0.05. We contacted with the coaching staffs of two teams of provincial level to inviting all their team members to participate in this study. Therefore, a total of 21 adolescent and 27 adult female soccer players were recruited from the two teams. The adolescents and adults Their demographic information is presented in Table 1. Participants were provided with course credit or money as a reward for participation. All participants had a self-reported normal vision and signed a written informed consent form. All subjects gave their informed consent for inclusion before they participated in the study. For adolescents, written informed consents were signed from their parents for study participation. The study was conducted in accordance with the Declaration of Helsinki, and the protocol was approved by the Ethics Committee of Shaanxi Normal University (No. 202316003).

**Table 1:** Demographic information of participants.

Variables	Adolescents (U15)	Adults (U23)
Age (by years, *M* ± *SD*)	13.50 ± 0.33	20.65 ± 1.22
Soccer Experience (by years, *M* ± *SD*)	4.76 ± 1.44	7.66 ± 2.25
Training per week (by hours, *M* ± *SD*)	9.00 ± 1.10	8.33 ± 1.24
Height (by cm, *M* ± *SD*)	163.14 ± 6.02	166.26 ± 5.40
Weight (by kg, *M* ± *SD*)	52.19 ± 6.88	55.70 ± 7.51
N (Total)	21	27

### Stimuli and materials

The present experiment was implemented and executed using Python (Python Software Foundation, version 3.7.3) and was performed on a 27-in. desktop display monitor (279M1RVE, PHILIPS, Netherlands) set with a resolution of 1280 × 720 and a refresh rate of 60 Hz. The stimuli were presented to participants on a monitor placed approximately 80 cm away, resulting in a visual angle of 42°.

#### Selection of video segments

The pattern anticipation task assessed participants’ ability to anticipate player field locations from briefly presented soccer match video segments. Three men Asian Football Confederation (AFC) Level A certified experienced soccer coaches selected video segments from men’s UEFA Champions League and top five European soccer leagues’ TV broadcasts. A total of 23 video segments were selected based on the criterion of including a decisive moment (i.e., the onset of a shot, pass, or dribble)^34^. These 23 video segments were then edited to an episode of 8-12 s to make it stop at the frame of decisive moments that were checked and selected by the three soccer coaches. Afterwards, two other men AFC Level A certified soccer coaches evaluated the 23 selected video segments for their offensive processes. They rated each video on a 10-point scale for its offensive processes, where 1 indicated a typical counter attack and 10 indicated a typical positional attack. Based on Sherwood’s et al.^30^ criterion on structured stimuli of pattern recall rated by two expert coaches (structured stimuli were rated 8–10 points and unstructured stimuli were rated 1–2 points), two other expert coaches agreed on 6 video segments of counter attacks (rated 8–10) and 5 video segments of positional attacks (rated 1–2). The video segments of both offensive processes presented positional attacks at the beginning. The offensive players in video segments of positional attacks completed all actions. In other word, we require participants not to consider the potential faults (e.g. intercept by opponents) made by offensive players in the video segments after decisive moments. However, in the counterattack video segments, the previous defensive players gained possession of the ball in the middle of the video segments and became offensive players. Finally, the time point of future location was selected by the overall AFC Level A coaches’ panel and varied depending on the pattern presented in soccer sports in line with Farrow et al.^20^. The criterion is that the soccer ball moves to a designated spot (1–2 s later until the action is complete), such as when the soccer ball is passed long or short from one player to another player.

All video segments were edited with Format Factory™, a free video editing software. The first two video segments (not rated) served as a practice block to familiarize observers with the task. A total of 11 video segments in the formal experimental block were analyzed.

### Task procedure

**Figure 1:**
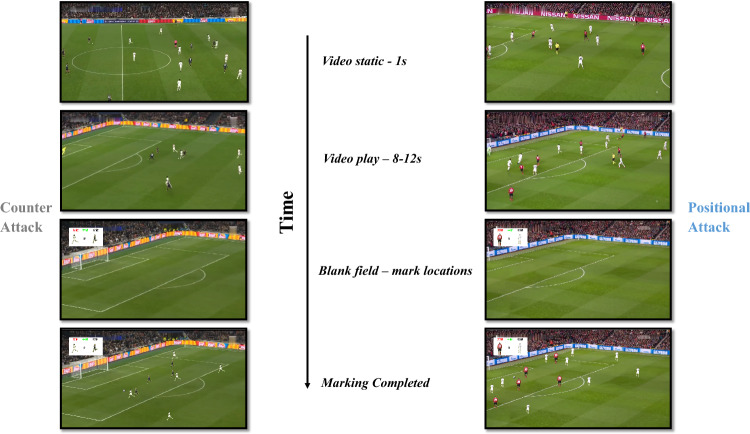
The chart above describes the procedure and offensive processes of the pattern anticipation task. The 11 video segments (six for counter attacks and five for positional attacks) were randomly presented to each participant. After a one-second pause, the video segments were displayed for approximately 8–12 s. Then, the video was stopped and a blank response image was shown. Participants were instructed to use a mouse to indicate future locations (approximately 1–2 s in the future, depending on whether players in the video completed their current action). Finally, the future location of the soccer ball and all offensive and defensive players were marked on the blank response image. The task took about 30 min on average for each participant.

The soccer match video segments were presented on a desktop display monitor. Participants observed the movement and location of all players except the goalkeepers and referees. There was a one-second static before the video played, followed by the video segments playing for about 8-12 s. When the video segments stopped, a blank soccer field response image with the same perspective as the original video segment appeared. Participants were asked to assume that the future action of the offensive side was completed correctly^20^, and then anticipated the future locations of the soccer ball and all players (except goalkeepers) from both teams on the response image (Fig. 1). The image removed players from both sides, the soccer ball, referees, and goalkeepers, but field markings and surrounding stadium structures were retained for spatial orientation.

In the anticipation phase, participants used the left and right mouse buttons to mark the location of players from both sides, and the middle mouse button to mark the location of the soccer ball. We used Adobe Photoshop™ to select one person from the offensive side and one from the defensive side as the team representative in each video segment. The teams represented by the left and right mouse buttons were displayed in the upper right corner of the screen. Clicking the corresponding button of the mouse would make a mark while pressing the button again at the same location would cancel the mark. When marking was completed, participants were required to press the SPACEBAR of the keyboard and received a notification asking them to verify whether the number of players was marked in accordance with the actual number of players in the video clips. If not, the program would prompt the difference and return to the answer interface until there was agreement.

Data were recorded in presentation of response images. The task program recorded the coordinates of the soccer ball, offensive players, and defensive players in future decisive moments for each video segment. A Python 3.7.3 analysis program pre-processed the original data to integrate correct answers and marked locations in the same blank field. The assessment matched the location answered by an observer only with the shortest correct location. The formula $\sqrt{{({x}_{1}-{x}_{2})}^{2}+{({y}_{1}-{y}_{2})}^{2}}$^19^ calculated radial error (RE) by the shortest location distance. The ${x}_{1}$ and ${y}_{1}$ indicates the participants' marked coordinates. The ${x}_{2}$ and ${y}_{2}$ indicates the elements’ (soccer ball and players) future coordinates in reality. The lower RE, the higher accuracy anticipated elements performed by participants. Then, average RE (aRE) was calculated, indicating average RE per every element (i.e., offensive or defensive players).

### Transparency and openness

We report how we determined our sample size, all data exclusions (if any), all manipulations, and all measures in the study, and we follow Journal Article Reporting Standards (JARS)^35^.

Data analyses were performed using IBM SPSS (Statistical Package for Social Sciences, version 26.0) software. This study’s design and its analysis were not pre-registered.

## Results

**Figure 2:**
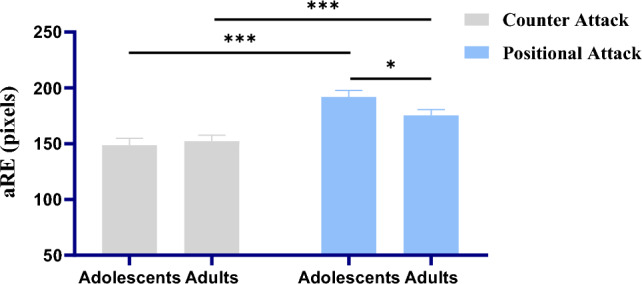
The chart above describes the interaction of offensive processes and age groups on average Radial Error (aRE). Error bars denote the standard error of the mean (SEM). The p-values were derived from post-hoc analysis (LSD) of interaction of offensive processes and age group after repeated-measurements ANOVA. *p < 0.05, **p < 0.01, ***p < 0.001.

The three-way within-between-subjects repeated-measurements ANOVA on aRE of pattern anticipation were analyzed. The 2 offensive processes (counter attacks, positional attacks) and 3 elements (soccer ball, offensive players, defensive players) are with-subjects independent variables, and 2 age groups (U15, U23) is between-subjects independent variable. There was a significant main effect of offensive processes (*F* (1, 46) = 56.823, *p* < 0.001, η_p_^2^ = 0.553), indicating offensive processes of counter attacks show significantly lower aRE than positional attacks. There was also a main effect of elements (*F* (2, 92) = 91.920, *p* < 0.001, η_p_^2^ = 0.666). After post-hoc analysis (Least Significant Difference (LSD) was used in all post-hoc analyzes in this study) of elements, we found anticipation of soccer ball (*M* = 212.73, *SE* = 7.15) was significantly higher in aRE than offensive players (*M* = 143.89, *SE* = 2.60; *p* < 0.001) and defensive players (*M* = 144.399, *SE* = 3.172; *p* < 0.001; Fig. 2), but offensive and defensive players were not significantly from each other in aRE (*p* = 0.824). The main effect of age groups was not significant (*F* (1, 46) = 0.959, *p* = 0.333, η_p_^2^ = 0.020).

The results of the three-way within-between-subjects repeated-measurements ANOVA on aRE of pattern anticipation showed a significant interaction between offensive processes and age groups (*F* (1, 46) = 5.282, *p* = 0.026, η_p_^2^ = 0.103). Post-hoc analysis indicated a significant aRE difference between adult and adolescent group in the offensive process of positional attacks (*M*_adolescent_ = 191.91, *SE* = 5.92; *M*_adult_ = 175.23, *SE* = 5.22; *p* = 0.040; Fig. 2), but not in the offensive process of counter attacks (*M*_adolescent_ = 148.67, *SE* = 6.12; *M*_adult_ = 152.20, *SE* = 5.40; *p* = 0.668).

**Figure 3:**
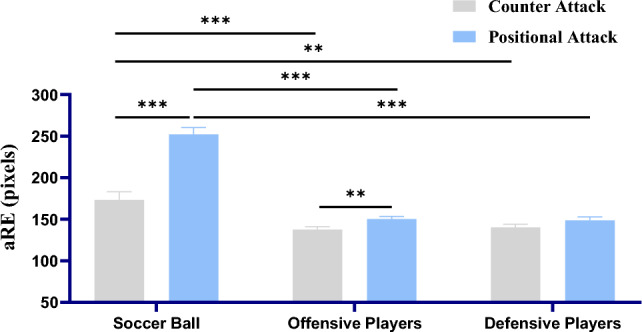
The chart above describes the interaction of offensive processes and elements on average Radial Error (aRE). Error bars denote the SEM. The p-values were derived from post-hoc analysis (LSD) of interaction of offensive processes and age group after repeated-measurements ANOVA. **p < 0.01, ***p < 0.001.

**Figure 4:**
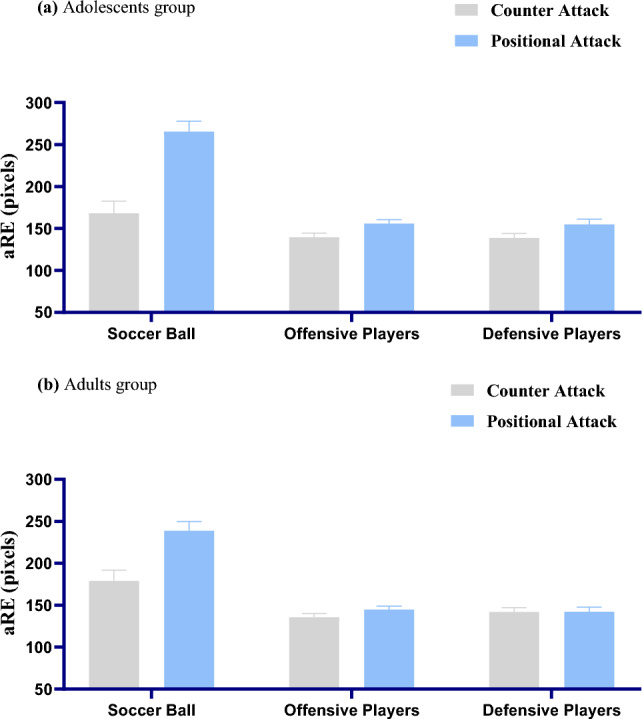
The chart above describes the interaction of offensive processes and elements on average Radial Error (aRE) between groups of adolescents (**a**) and adults (**b**).

There was also a significant interaction of offensive processes and elements (*F* (2, 92) = 29.591, *p* < 0.001, η_p_^2^ = 0.391). Post-hoc analysis indicated significant aRE difference between offensive processes while selecting soccer ball (*M*_counter attack_ = 173.40, *SE* = 9.74; *M*_positional attack_ = 252.05, *SE* = 8.31; *p* < 0.001; Fig. 3), and significant aRE difference between offensive processes while selecting offensive players (*M*_counter attack_ = 137.62, *SE* = 3.33; *M*_positional attack_ = 150.16, *SE* = 3.34; *p* = 0.004), but there was no significant difference in aRE between offensive processes when selecting defensive player (*M*_counter attack_ = 140.29, *SE* = 3.72; *M*_positional attack_ = 148.509, *SE* = 4.18; *p* = 0.089). Further, in counter attack condition, aRE of soccer ball was significantly higher than aRE of offensive players (*p* < 0.001) and defensive players (*p* = 0.002), but there was no significant aRE difference between offensive players and defensive players (*p* = 0.418; Fig. 3). Similarly, in positional attack condition, aRE of soccer ball was also significantly higher than aRE of offensive players (*p* < 0.001) and defensive players (*p* < 0.001), and there was also no significant aRE difference between offensive players and defensive players (*p* = 0.646; Fig. 3). There was no three-way interaction of age groups, offensive processes and elements significant ((*F* (2, 92) = 1.141, *p* = 0.324, η_p_^2^ = 0.024; Fig. 4).

## Discussion

In the present study, the pattern anticipation task in soccer was examined for two age groups of female players. The results showed that all age groups were more accurate in anticipating the soccer ball and offensive players during counter attacks than during positional attacks, but this was not observed when anticipating defensive players. Additionally, the anticipation accuracy for the soccer ball was lower than for the players, suggesting that the dynamic movement of the soccer ball is indeed challenging to anticipate. These findings support Hypothesis 2.

It was found that adult soccer players only performed better in anticipating locations during positional attacks. Furthermore, there was no overall better performance in anticipating offensive players in the adult group compared to adolescents. These results did not fully support Hypothesis 1. These findings may be related to the different cognitive processes involved in counter attacks and positional attacks among soccer players. Counter attacks are fast and direct attacks that occurs from a moment of transition^36^. It consists of minimal passes and aims to create a goal-scoring opportunity before the opposition can recover their defensive shape^37^. In contrast, positional attacks are more methodical approaches to creating a scoring opportunity^38^, refer to players coordinate their spatial positioning over time to achieve a common goal^27^. It involves building up play from the back, with the aim of creating space and opportunities to penetrate the opposition’s defense^1^. In both cases, teams interact with their opponents and constantly adapt to their movements. Positional attacks are considered more structured than counter attacks^39^. Thus, adult female soccer players with more technical and tactical experience than adolescents performed better in positional attack video segments with more structured presentation. These findings are consistent with Sherwood et al.^30^, who compared various levels of rugby players in a pattern recall task.

The results of the present study suggest that the accuracy of element anticipation by female soccer players varies during different offensive processes. The anticipation advantage during counter attacks was only evident for the soccer ball and offensive players, but not for defensive players. This result may indicate a tendency among female soccer players to neglect defensive players during counter attacks. In other words, due to fewer defensive players being present during counter attacks than during positional attacks, the running paths of offensive players may be less affected by defensive players. The success of a counter attack may be more closely associated with the location of offensive players and the soccer ball, thus influencing the perception of these elements. Previous studies have suggested that team sports players indeed allocate more attentional resources to anticipating the location of offensive players^16,18^, especially during decision-making processes^14^. The present study further supports this finding in relation to soccer ball location anticipation during different offensive processes.

Counter attacks and positional attacks are two main offensive processes in soccer sports. Due to a relatively low number of players participating in the counter attack process, there seems to remain a difficulty difference between these two offensive processes. However, based on the result of aRE difference between counter attacks and positional attacks, it was found that there was still no significant difference in aRE for defensive players, which also performed lower numbers of players in participating in counter attacks than positional attacks^40,41^. Additionally, players under counter attack offensive process usually move at a higher speed than those in positional attack process. Participants’ ability to track element motion speed is suggested to negatively correlate with the number of tracked elements^42^. Counter attacks with high-speed elements may require more task demand than positional attacks with low-motion speed. Conversely, positional attacks with a higher number of elements may also require more task demand to anticipate than counter attacks. In summary, the difficulty difference between counter attacks and positional attacks is similar but manifests differently, and thus may not affect the overall result of the present study.

This study addressed the previously neglected issue of soccer ball location. We analyzed the RE of soccer ball anticipation to evaluate its difference from the anticipation of offensive and defensive players. Anticipation of soccer ball and offensive players both performed better in offensive processes of counter attacks than positional attacks. The moving elements (players) in video segments generally follow structured paths with formations such as 4-4-2, 4-3-3, and 4-5-1^43^. The anticipation score of the soccer ball may affect overall performance in pattern recognition because it is closely associated with soccer tactics and formation^28,44^. Therefore, anticipating players’ locations during a game may be strongly related to anticipating the location of the soccer ball. In other words, perceiving the location of soccer ball may serve as a fundamental area of interest of perceiving location of the players. This study provides preliminary work on this issue and we recommend that future studies on perceptual-cognitive skills in team sports consider the role of ball location. A larger aRE in soccer ball anticipation than both offensive and defensive player indicates the relative high demand to anticipate soccer ball location. One potential reason is the rapid moving speed and small visual angles of soccer ball presented on the screen. Moving speed and visual angles are suggested affect tracking multiple moving objects^45^. Apart from this reason, sidespin while soccer ball moving also add its location anticipation difficulty for participants. The video segments were stop at the decisive frame indicates the proximity of shots, passes, or dribbles. This also indicates the dynamically potential movement of soccer ball. In the presence of spin, the resulting ball trajectory systematically reduced the judgments made by both expert and non-soccer-players^46^.

In this study, it was found that adult female soccer players anticipated locations with higher accuracy than adolescents during positional attacks. Female soccer players also demonstrated a higher anticipation accuracy towards the soccer ball and offensive players during counter attacks than during positional attacks. This finding suggests that positional attack is the one of the specific advantages in perceptual-cognitive skills for adult female soccer players. Additionally, the anticipation of elements in perceptual-cognitive skills for female soccer players varied between offensive processes.

There are some practical applications for coaches and other team staffs in soccer teams. Enhancing the perceptual-cognitive skills of adolescent female soccer players in positional attacks is worth considering in normal training. Coaches may consider increasing the percentage of tactics training in positional attacks. Additionally, coaches may take pattern anticipation tasks as a cognitive training method combined with normal tactics or physical training, enhancing the anticipation skills in positional attacks for adolescent female soccer players. When selecting talents for future training, it is important to consider the essential role of offensive processes and distinguish between selected elements (soccer ball and players) when measuring perceptual-cognitive skills like anticipation.

## Limitations and future studies

First, the difficulty difference between counter attacks and positional attacks is similar but manifests differently in varied element number and element motion speed. Future study should address this by adopting more fine experimental design than current study. Second, eye-tracking measurements for pattern anticipation tasks can address the varied perceptual tendencies towards the soccer ball and offensive and defensive players during different offensive processes. This is an important area for future research. Third, the third-person perspective of a video-based pattern anticipation task may differ from the first-person perspective experienced during a real game situation. Without the use of virtual reality technology, it is difficult to capture a realistic viewing perspective that allows players to fully explore their performance environment. Therefore, future studies could utilize pattern anticipation task under virtual reality conditions with a first-person perspective to enhance ecological validity.

## Data Availability

Data is applicable on 10.6084/m9.figshare.24131685.

## References

[1] Lago C, Martín R (2007). Determinants of possession of the ball in soccer. J. Sports Sci..

[2] Mann DTY, Williams AM, Ward P, Janelle CM (2007). Perceptual-cognitive expertise in sport: A meta-analysis. J. Sport Exerc. Psychol..

[3] Casanova F (2013). Effects of prolonged intermittent exercise on perceptual-cognitive processes. Med. Sci. Sports Exerc..

[4] Scharfen H-E, Memmert D (2019). Measurement of cognitive functions in experts and elite athletes: A meta-analytic review. Appl. Cogn. Psychol..

[5] Voss MW, Kramer AF, Basak C, Prakash RS, Roberts B (2010). Are expert athletes ‘expert’ in the cognitive laboratory? A meta-analytic review of cognition and sport expertise. Appl. Cogn. Psychol..

[6] Machado G, Da Costa IT (2020). TacticUP video test for soccer: Development and validation. Front. Psychol..

[7] Che, X. *et al.* Two-dimensional and three-dimensional multiple object tracking learning performance in adolescent female soccer players: The role of flow experience reflected by heart rate variability. *Physiol. Behav.* **258**, 114009. 10.1016/j.physbeh.2022.114009 (2023). 10.1016/j.physbeh.2022.114009 36326537

[8] Schumacher N, Schmidt M, Wellmann K, Braumann K-M (2018). General perceptual-cognitive abilities: Age and position in soccer. PLoS ONE.

[9] Murr D, Larkin P, Höner O (2021). Decision-making skills of high-performance youth soccer players. Ger. J. Exerc. Sport Res..

[10] Vilar L, Araújo D, Davids K, Bar-Yam Y (2013). Science of winning soccer: Emergent pattern-forming dynamics in association football. J. Syst. Sci. Complex.

[11] Roca A, Ford PR, Memmert D (2021). Perceptual-cognitive processes underlying creative expert performance in soccer. Psychol. Res..

[12] de Waelle S, Warlop G, Lenoir M, Bennett SJ, Deconinck FJA (2021). The development of perceptual-cognitive skills in youth volleyball players. J. Sports Sci..

[13] Schapschröer M, Baker J, Schorer J (2016). Exploring the interaction of physical exercise load and pattern recall performance in female handball players. Exp. Brain Res..

[14] Gorman AD, Abernethy B, Farrow D (2015). Evidence of different underlying processes in pattern recall and decision-making. Q. J. Exp. Psychol..

[15] North JS, Hope E, Williams AM (2016). The relative importance of different perceptual-cognitive skills during anticipation. Hum. Mov. Sci..

[16] Gorman AD, Abernethy B, Farrow D (2012). Classical pattern recall tests and the prospective nature of expert performance. Q. J. Exp. Psychol..

[17] Ward P, Williams AM (2003). Perceptual and cognitive skill development in soccer: The multidimensional nature of expert performance. J. Sport Exerc. Psychol..

[18] van Maarseveen MJJ, Oudejans RRD, Mann DL, Savelsbergh GJP (2018). Perceptual-cognitive skill and the in situ performance of soccer players. Q. J. Exp. Psychol..

[19] Gorman AD, Abernethy B, Farrow D (2013). The expert advantage in dynamic pattern recall persists across both attended and unattended display elements. Atten. Percept. Psychophys..

[20] Farrow D, McCrae J, Gross J, Abernethy B (2010). Revisiting the relationship between pattern recall and anticipatory skill. Int. J. Sport Psychol..

[21] Kalén A (2021). The role of domain-specific and domain-general cognitive functions and skills in sports performance: A meta-analysis. Psychol. Bull..

[22] Abernethy, B., Farrow, D. & Mann, D. L. In *The Cambridge Handbook of Expertise and Expert Performance,* (eds Ericsson, K. A. *et al.*) 677–695 (Cambridge University Press, 2018).

[23] McRobert AP, Ward P, Eccles DW, Williams AM (2011). The effect of manipulating context-specific information on perceptual-cognitive processes during a simulated anticipation task. Br. J. Psychol..

[24] Abernethy B, Gill DP, Parks SL, Packer ST (2001). Expertise and the perception of kinematic and situational probability information. Perception.

[25] Sarmento H, Anguera T, Campaniço J, Leitão J (2010). Development and validation of a notational system to study the offensive process in football. Medicina.

[26] Tenga A, Sigmundstad E (2011). Characteristics of goal-scoring possessions in open play: Comparing the top, in-between and bottom teams from professional soccer league. Int. J. Perform. Anal. Sport.

[27] Low B (2020). A systematic review of collective tactical behaviours in football using positional data. Sports Med..

[28] Niu Z, Gao X, Tian Q (2012). Tactic analysis based on real-world ball trajectory in soccer video. Pattern Recogn..

[29] Gorman AD, Abernethy B, Farrow D (2013). Is the relationship between pattern recall and decision-making influenced by anticipatory recall?. Q. J. Exp. Psychol..

[30] Sherwood S, Smith T, Masters RSW (2019). Pattern recall, decision making and talent identification in rugby union. Eur. J. Sport Sci..

[31] Raab M, Farrow D (2015). Examining the stability and specificity of pattern recall in team handball. Int. J. Sport Psychol..

[32] Huijgen BCH (2015). Cognitive functions in elite and sub-elite youth soccer players aged 13 to 17 years. PLoS ONE.

[33] Fernandez-Navarro J, Fradua L, Zubillaga A, Ford PR, McRobert AP (2016). Attacking and defensive styles of play in soccer: Analysis of Spanish and English elite teams. J. Sports Sci..

[34] van Maarseveen MJJ, Oudejans RRD, Savelsbergh GJP (2015). Pattern recall skills of talented soccer players: Two new methods applied. Hum. Mov. Sci..

[35] Kazak AE (2018). Editorial: Journal article reporting standards. Am. Psychol..

[36] Hewitt A, Greenham G, Norton K (2016). Game style in soccer: What is it and can we quantify it?. Int. J. Perform. Anal. Sport.

[37] Gómez M-Á, Mitrotasios M, Armatas V, Lago-Peñas C (2018). Analysis of playing styles according to team quality and match location in Greek professional soccer. Int. J. Perform. Anal. Sport.

[38] Bate R, Reilly T, Lees A, Davids K, Murphy WJ (2018). Football chance: Tactics and strategy. Science and Football.

[39] Goes FR, Brink MS, Elferink-Gemser MT, Kempe M, Lemmink KAPM (2021). The tactics of successful attacks in professional association football: Large-scale spatiotemporal analysis of dynamic subgroups using position tracking data. J. Sports Sci..

[40] Fernandez-Navarro J, Fradua L, Zubillaga A, McRobert AP (2019). Evaluating the effectiveness of styles of play in elite soccer. Int. J. Sports Sci. Coach..

[41] Asian-Clemente JA, Rabano-Muñoz A, Suarez-Arrones L, Requena B (2023). Different pitch configurations constrain the external and internal loads of young professional soccer players during transition games. Biol. Sport.

[42] Alvarez GA, Franconeri SL (2007). How many objects can you track? Evidence for a resource-limited attentive tracking mechanism. J. Vis..

[43] Bradley PS (2011). The effect of playing formation on high-intensity running and technical profiles in English FA Premier League soccer matches. J. Sports Sci..

[44] Chung D, Carvalho T, Casanova F, Silva P (2019). Number of players manipulation effect on space and concentration principles of the game representativeness during football small-sided and conditioned games. JPES.

[45] Fehd HM, Seiffert AE (2010). Looking at the center of the targets helps multiple object tracking. J. Vis..

[46] Craig CM (2009). Optic variables used to judge future ball arrival position in expert and novice soccer players. Atten. Percept. Psychophys..

